# Epidermal cyst formation of the lip following autologous fat transplant

**DOI:** 10.1016/j.jdcr.2022.06.024

**Published:** 2022-07-02

**Authors:** Patricia Monnet, Martine Bagot, Jacqueline Rivet, Jennifer Roux, Antoine Petit, Jana Al-Hage

**Affiliations:** a Department of Dermatology, Saint-Louis Hospital, Assistances Publiques des Hôpitaux de Paris, Paris, France; b Université Paris Cité, Paris, France; c Department of Pathology, Saint-Louis Hospital, Assistances Publiques des Hôpitaux de Paris, Université Paris Cité, Paris, France

**Keywords:** autologous fat injections, autologous fat transplant, autologous fat transfer, epidermal cyst, epidermoid cyst, fat grafting, fillers, keratin cyst, lipofilling, AFT, autologous fat transfer

## Introduction

Epidermal cysts are derived from the follicular infundibulum and are exceptionally found over glabrous skin, including the lips and oral mucosa. To date, only approximately 10 cases of acquired epidermal cysts of the buccal mucosa have been reported in the literature without a clear etiology. ^1^ We, here, report another unusual case of an epidermal cyst formation on the lip that might have developed following an autologous fat transplant, giving possible explanations about its etiopathogenesis.

## Case report

A 53-year-old white man presented to our dermatology department for an infiltrated asymptomatic nodule of the left side of the upper lip. His medical history included HIV infection, kidney transplantation, cardiovascular disease, and an autologous fat transfer (AFT) done for cosmetic upper lip augmentation 4 years ago. The nodule appeared a few months following the AFT and has been growing progressively since then. He did not recall any other inciting trauma. Details about the lipofilling procedure were not available.

**Fig 1:**
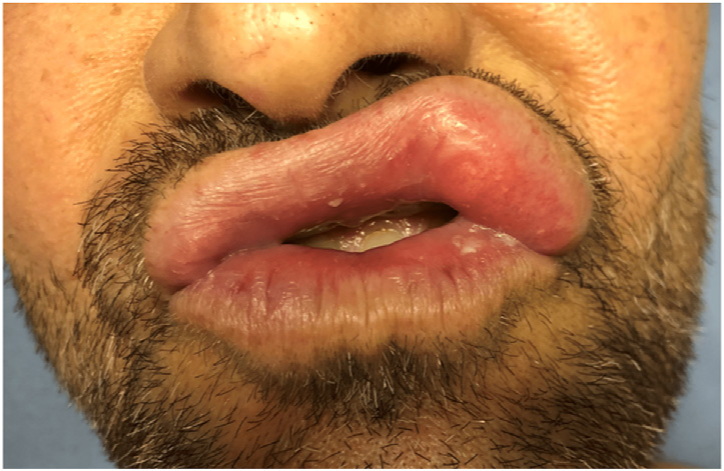
Initial presentation with a firm indurated nontender nodule of the left side of the upper lip.

Physical examination revealed an indurated ill-defined nodule of the left side of the upper lip, resulting in labial asymmetry (Fig 1). Differential diagnoses included orofacial granulomatosis, foreign body granulomatous reaction, fat hypertrophy, or a salivary gland tumor. An ultrasound showed a 15-mm echogenic lipomatous mass.

**Fig 2:**
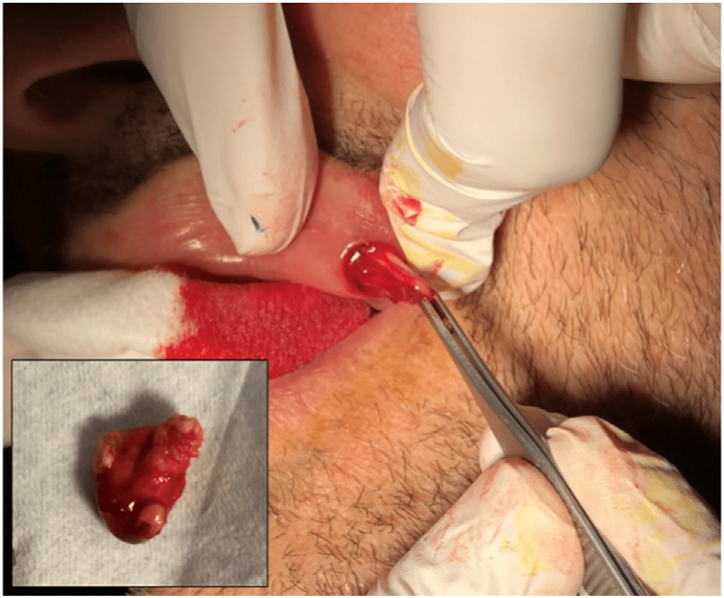
Punch excision through an intraoral mucosal approach with drainage of the autologous fat tissue and enucleation of a cystic lesion. Accidental partial cystic wall rupture shows evidence of cheesy content.

A punch incision was performed under local anesthesia through the upper labial mucosa for tissue sampling (Fig 2).

**Fig 3:**
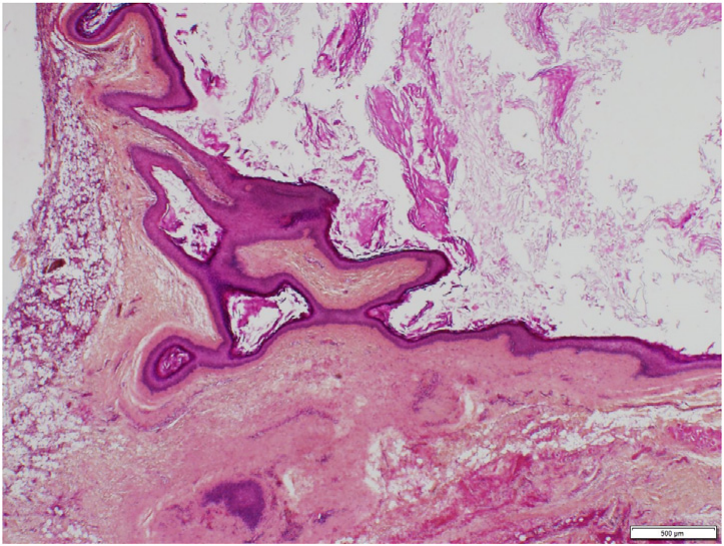
Histopathologic examination revealed a cystic lesion with a keratinized stratified squamous epithelium filled with amorphous lamellar keratin debris. The cyst is surrounded by fibrosis and clumps of mature adipocytes. (Hematoxylin-eosin stain; original magnification: ×4.)

Interestingly, an amorphous lipomatous yellowish material was extracted, along with an underlying cystic lesion of approximately 1 cm in diameter. A partial rupture of the cystic wall accidentally occurred, with drainage of a cheesy-like white keratin content. The entire specimen was sent for histopathologic evaluation. Microscopically, the specimen was composed of mature adipocytes surrounding an epidermal keratin cyst (Fig 3).

**Fig 4:**
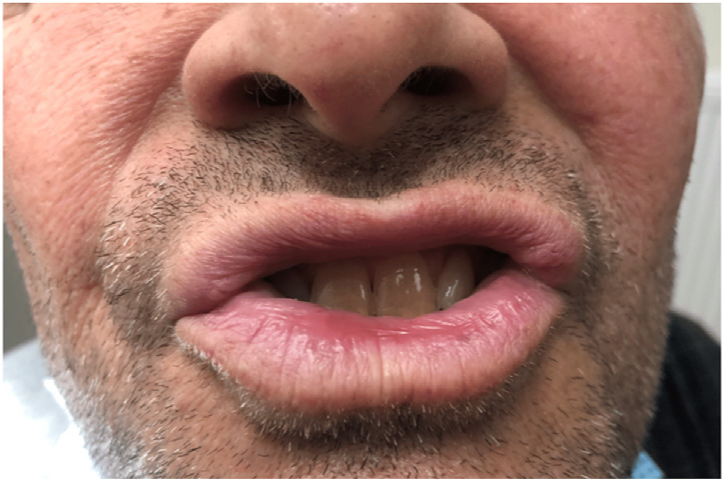
Final clinical appearance, 1 month after the procedure.

After 30 days of follow-up, the patient was satisfied with the result (Fig 4). At 1-year follow-up, there was no evidence of recurrence.

## Discussion

AFT, also known as lipofilling, is increasingly being used in cosmetic and reconstructive procedures of the face, essentially for facial rejuvenation, and to correct atrophic conditions.^2^ This technique has the advantage over other dermal fillers of being safe because of the biocompatibility of adipose tissue. Complications are rare and include asymmetry, prolonged edema, infections at the injection site or the harvest site, poor fat viability and fat necrosis, graft hypertrophy, and even more serious complications such as vascular embolism when injected in the glabella or temple.^3^

Pseudocysts formation or “oil cysts” have been reported with AFT, mainly with breast surgery and exceptionally following AFT of the face.^4^ These pseudocysts do not have a true cystic wall and are distinct from epidermal cysts; they result from fat necrosis and may depend on the method of fat purification and the vascular supply. Another rare type of cystic lesions has also been reported with other dermal fillers such as silicone injections and hyaluronic acid fillers but not with AFT; these cysts result from inflammation, encapsulation, and the formation of a fibrous capsule trapping the filling material.^5^ However, to the best of our knowledge, epidermal keratin cyst formation has never been reported after AFT or with any other dermal filler injections.

On the other hand, epidermal cysts are extremely rare on hairless skin, especially on the lips and oral mucosa. Yet, few cases have been reported in the literature without any clear etiology.^1^ The proposed mechanism for the formation of such cysts was that of a posttraumatic migration and “implantation” of the keratinizing epidermis into deep tissues; however, a clear injury preceding the formation of the cyst was almost never evident.

A lip cyst developed in our patient a few months after having undergone an AFT. This striking occurrence may be by mere coincidence but could also straightforwardly reinforce the theory of implantation of keratinizing epidermis that may develop during the AFT procedure. The grafted fat tissue itself may also potentially play a role in the induction or progression of this cyst, depending on its quality and its stem cell content, but further similar cases are needed to confirm this theory.

In conclusion, we report a rare case of an acquired epidermal cyst of the lip as a possible complication of AFT. This finding may confirm the hypothesis of a posttraumatic origin of epidermal inclusion of keratin cysts over the buccal mucosa. In this case, a less traumatic technique of AFT might be considered. Treatment of these cysts is surgical and best done with a punch excision and enucleation via an intraoral approach.

## Conflicts of interest

None disclosed.
